# Telediagnosis of dental caries: Possible or impossible? A pilot cross‐sectional study

**DOI:** 10.1002/cre2.663

**Published:** 2022-09-22

**Authors:** Francesca Zotti, Luca Rosolin, Francesco Simoncelli, Davide Pappalardo, Annalisa Cominziolli, Nicoletta Zerman

**Affiliations:** ^1^ Department of Surgical Sciences, Paediatrics and Gynecology University of Verona Verona Italy; ^2^ Private Practice in General dentistry in Verona Verona Italy; ^3^ Private Practice in General Dentistry in Padova Padua Italy

**Keywords:** dental caries, diagnosis, oral diagnosis, telemedicine

## Abstract

**Objectives:**

The aims of this study were to evaluate the effectiveness of teledentistry (based on a home intraoral imaging protocol) in detecting dental caries and to assess the accuracy of this method compared to clinical examination.

**Methods:**

Forty‐three patients were recruited for the study. Using a protocol for taking intraoral photographs at home with a smartphone proposed by the Dental School of Verona, a remote diagnosis of dental caries (TD) was performed by an experienced dentist. The same caries sites were also assessed by clinical diagnosis (CD) by a second experienced dentist. Ten photos were taken at home in five different perspectives, with and without flash, and emailed to one of the authors. The best five photos were selected for telediagnosis. The International Caries Detection and Assessment System (ICDAS II) score was used for caries diagnosis. Statistical tests were performed: Sensitivity and specificity of TD, the positive and negative predictive value of TD (PPV–NPV), and Spearman correlation to evaluate the relationship between the scores of TD and CD.

**Results:**

A total of 430 photographs were submitted; TD was performed on 215 photographs and 43 patients were visited. A total of 1201 teeth were analyzed. The sensitivity of TD was 74.0, the specificity was 99.1, the PPV of TD was 91.7, and the NPV was 96.4. The Spearman correlation was 0.816, showing a very strong correlation between the values obtained with TD and CD.

**Conclusions:**

The study showed good potential for TD, which proved to be a feasible method to combine with routine caries diagnosis in daily preventive dentistry practice.

## 1 INTRODUCTION

Telemedicine is an advancement of medicine that makes use of technology and technological tools. This concept has given rise to telediagnosis, which helps us to identify possible diseases and assess them remotely. Telemedicine provides care, consultation, and treatment based on telematic communication between patient and doctor (Ekeland et al., 2010). It seems to bring real benefits in certain cases, such as in economically less well‐developed countries where preliminary triage of patients could be very useful to save money and time. Telediagnosis could also help in early diagnosis and referral in countries where health facilities are not easily accessible (Mahdi et al., 2022). The current literature highlights the potential of telemedicine, which has been used particularly in dermatology, neurology, otolaryngology, and in oral pathology (May et al., 2008; Sakar & Kursun, 2010; Zhang, 2017). There have been some successes in mapping nevi or detecting precancerous lesions, diagnosing Parkinson's disease, and endoscopy of the ears and nose (Sakar & Kursun, 2010; Wu et al., 2014). Telemedicine can be applied in three ways: videoconferencing, remote monitoring, and remotely monitored treatment or education. Video conferencing between patients and physicians allows the two individuals to be connected for a consultation. Remote monitoring of patients' conditions can be performed using surveys, physiological sensors, or diaries. Remotely monitored treatment or education is targeted at groups of people in remote villages to improve their quality of life (Klaassen et al., 2016). Smartphones and smart devices are widely used in people's lives. Health applications such as “Apple Health” (Apple Inc.) are changing our medical consultation process; everyone has a clinical diary and a strong health motivation in their pocket (Zotti et al., 2016; Zotti, Pietrobelli, et al., 2019; Zotti, Zotti, et al., 2019). Leão and Porter (1999) reported that the potential of the Internet for remote diagnosis of oral diseases had not yet been explored, although the number of computer applications in dentistry has grown impressively.

Nowadays, remote diagnosis is widely used in preventive dentistry to detect precancerous oral lesions, oral diseases, and periodontal diseases and provide patient referrals and increase motivation and adherence to care (Khan & Omar, 2013; Zotti et al., 2016; Zotti, Zotti, et al., 2019). This practice resulted in a 50% reduction in associated costs, particularly related to unnecessary patient referrals for oral surgery emergencies (Roccia et al., 2005). Kopycka‐Kedzierawski and colleagues conducted a series of studies looking at the role of teledentistry in the detection and diagnosis of early childhood caries through intraoral imaging. The results of their work suggest that teledentistry could be a potentially valuable tool for caries screening (Kopycka‐Kedzierawski et al., 2008; Kopycka‐Kedzierawski & Billings, 2006, 2011). This study aims to evaluate the effectiveness of teledentistry in detecting dental caries. Specifically, the aims of this study were:

1. evaluating the feasibility of remote diagnosis of dental caries using intraoral photographs taken at home and
2. evaluating the accuracy of telediagnosis of dental caries compared to routine clinical diagnosis (CD) at the dental chair.

## 2 METHODS

The present observational study was conducted in accordance with the strengthening of the reporting of observational studies in epidemiology statement and was conducted as follows:

1. CD of dental caries at the dental chair by an experienced dentist (Clinician 1);
2. telediagnosis of dental caries (TD) by an experienced dentist (Clinician 2) using intraoral photographs taken at home with a smartphone showing the occlusal and vestibular surfaces of the upper and lower dental arches.

### 2.1 Protocol for taking appropriate intraoral home photographs and required photographs

The authors previously established a protocol for intraoral home photographs to be used for this study. The most important purpose of these photographs was to be able to see as large a tooth surface as possible, as well as all teeth in both arches (except wisdom teeth), and to minimize light effects.

The photographs, useful for remote diagnosis, must capture the dental arches as well as the occlusal and vestibular surfaces and provide images from which a clinician can assess the presence or absence of lesions.

The required parameters were:

− 8 Mpx camera in smartphone with built‐in flash;
− sufficient ambient lighting to capture all vestibular tooth surfaces without a flash;
− the sitting position and the inclination of the participant's head to both dental arches could be photographed (individually) with a suitable focus;
− the photographs should be taken by a nonprofessional photographer (a parent, friend, or relative, of course, and a lay person who can use a smartphone to take photographs); and
− the ability of the photographed participant to help the photographer avoid disturbing the soft mouth tissue in the picture.

**FIGURE 1:**
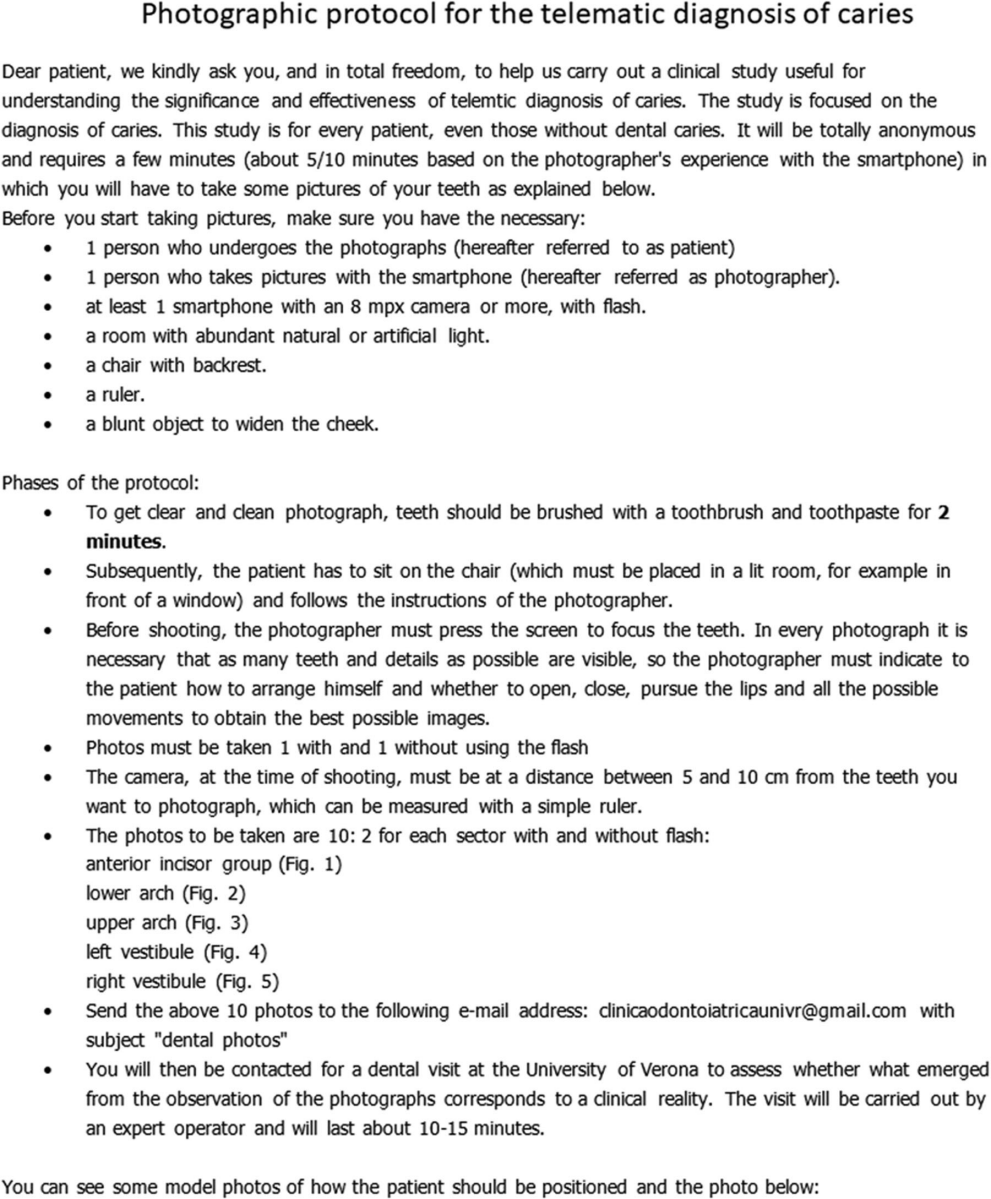
Flyer for displaying explanatory and descriptive information for the making of photographs and to clarify the objects of our project.

To help subjects learn how to take photographs, volunteers were given a leaflet with explanatory and descriptive information (Figure 1).

**FIGURE 2:**
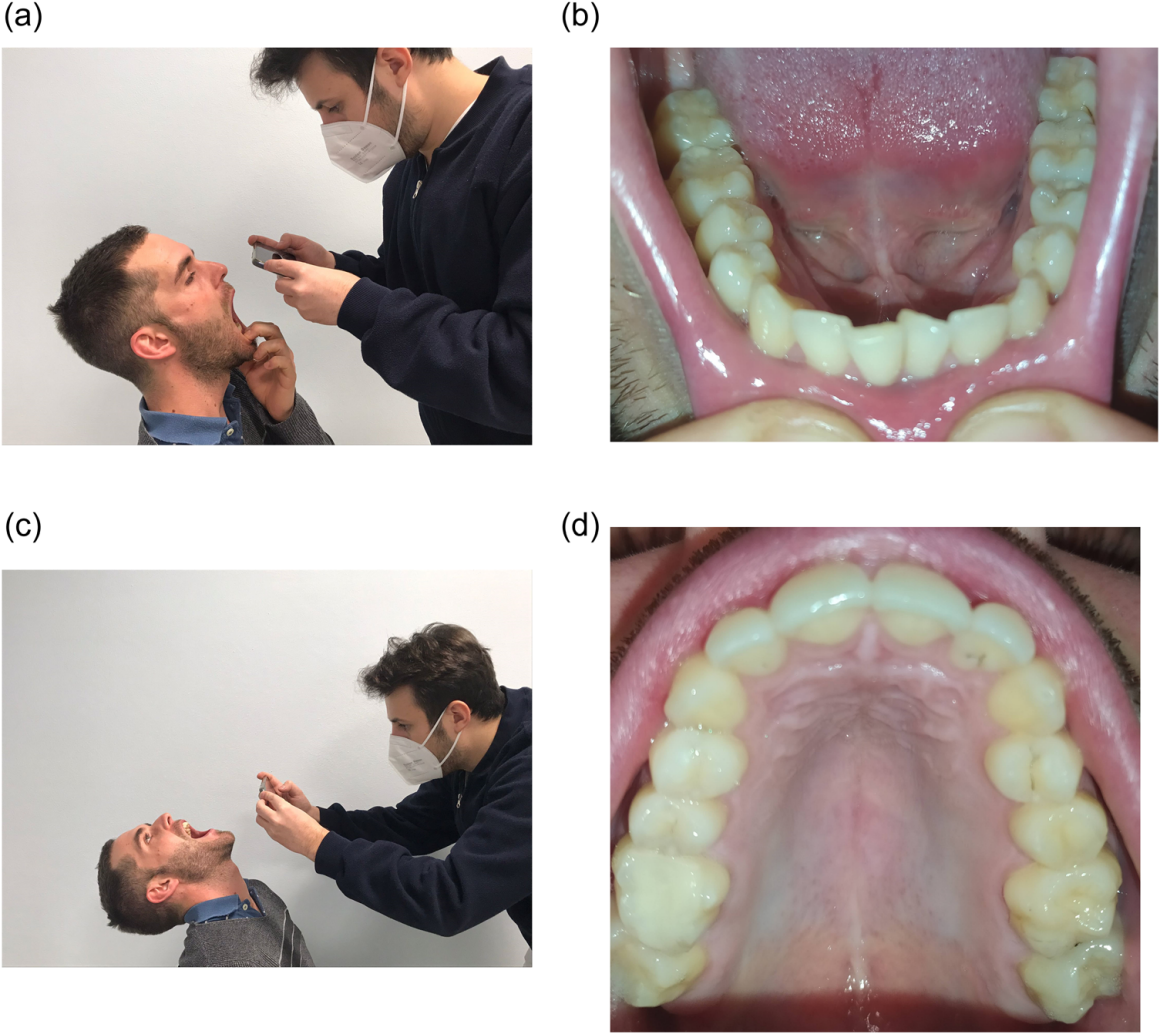
(a) Positioning for the lower arch, (b) photograph of the lower arch, (c) positioning for the upper arch, and (d) photograph of the upper arch.

**FIGURE 3:**
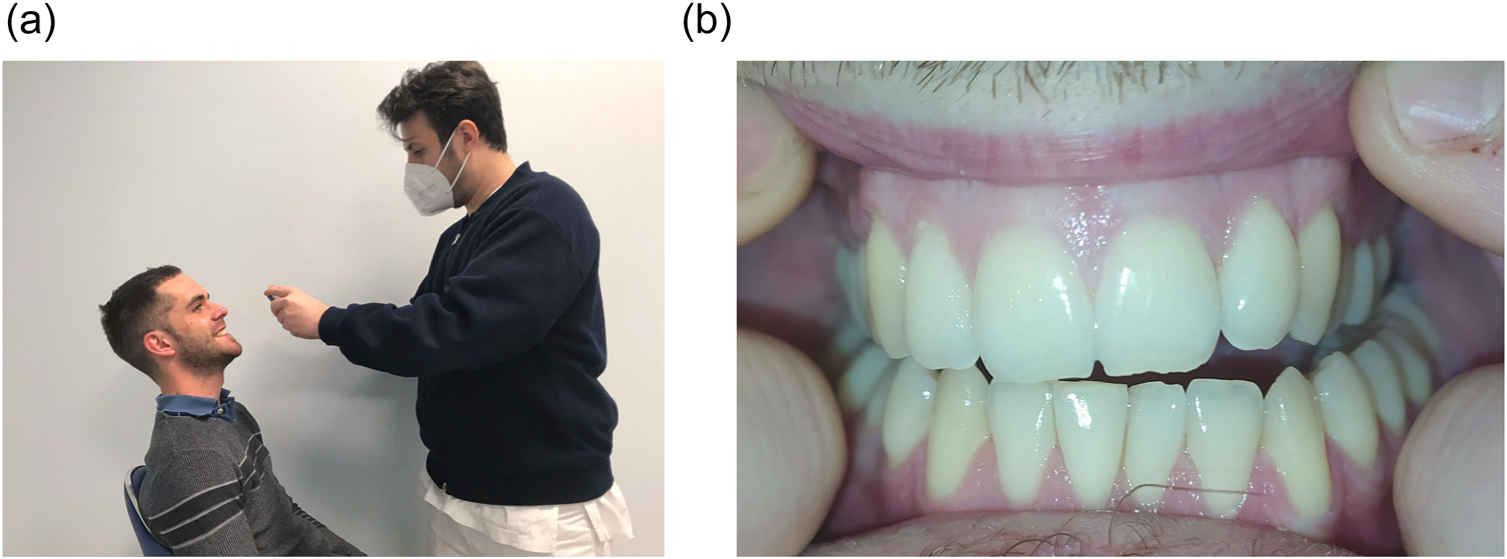
(a) Position for the frontal vestibular sector and (b) photograph of the frontal vestibular sector.

**FIGURE 4:**
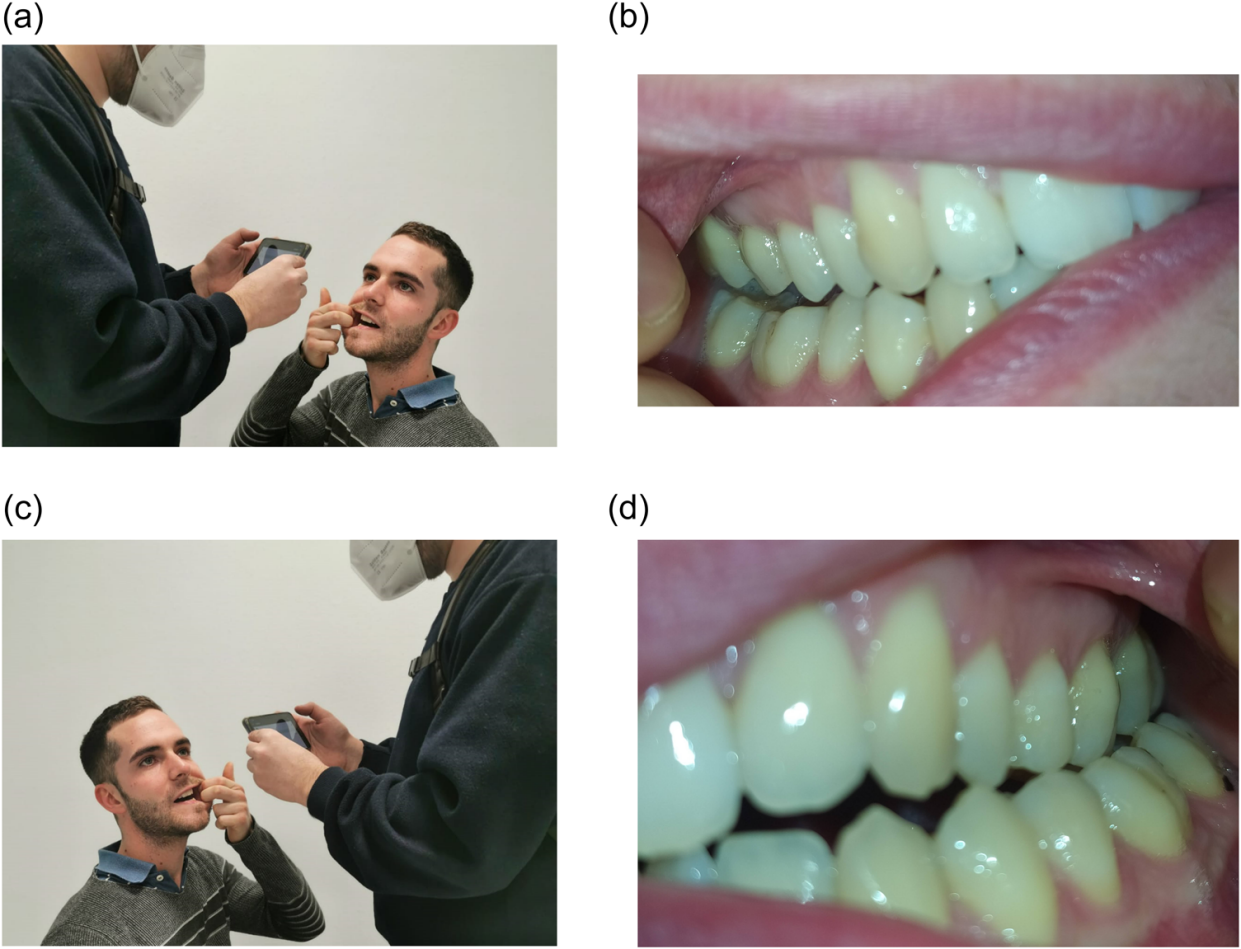
(a) Position for right vestibule, (b) photograph of right vestibule, (c) position for left vestibule, and (d) photograph of left vestibule.

Each participating volunteer was asked to take 10 pictures as follows:

− of the occlusal surface of the lower arch (with and without flash) (Figure 2a,b);
− 2 of the occlusal surface of the upper arch (with and without flash) (Figure 2c,d);
− 2 of the frontal vestibular sector, upper and lower view (with and without flash) (Figure 3a,b);
− 2 of the right vestibular surfaces (with and without flash) (Figure 4a,b); and
− 2 of the left vestibular surfaces (with and without flash) (Figure 4c,d).

One of the authors set up a special email box to receive all the patients' photos. The same author stored them and classified each set of photos with an alphanumeric code to preserve the anonymity of the participants. Patients were prohibited from sharing photos or sending text messages that could reveal they are true identities. Each participant was given an alphanumeric code, which was also transmitted to the two clinicians who conducted the CD and TD. No sample size calculation was done as there are no previous studies on this topic in the literature. Furthermore, participation in this study was voluntary and the enrolled participants were all students of the Faculty of Dentistry at the University of Verona, Italy.

The inclusion criteria for participation in the study were:

− Owners of a smartphone with an 8 Mpx camera and built‐in flash;
− a parent/relative/friend able to take intraoral photos with a smartphone;
− sufficient mouth opening (at least 35 mm); and
− possession of an email inbox and a suitable internet connection to send the photos.

Exclusion criteria were:

− Suffering from a systemic illness or eating disorders;
− taking medication or therapy; and
− vegan diet (Laffranchi et al., 2010).

### 2.2 CD of dental caries at the dental chair

This phase was a routine chairside examination conducted by an experienced dentist (Clinician 1). A dental explorer and airflow were used, avoiding magnification systems.

All tooth elements, except wisdom teeth, were examined for occlusal and vestibular cavities. The interproximal lesions were excluded from caries detection (Poorterman et al., 2000). International Caries Detection and Assessment System (ICDAS II) was used for caries diagnosis (Dikmen, 2015).

This index is a clinical scoring system that allows the detection and assessment of caries activity. For our study, the following classification (scores) was used:

− 0: inconspicuous;
− 1: first visible change in the tooth enamel;
− 2: clear visual change in enamel;
− 3: localized enamel loss (without clinical visual signs of dentin involvement);
− 4: underlying dark shadow of dentin;
− 5: clear cavity with visible dentin; and
− 6: extensive clear cavity with visible dentin.

Presence/absence of lesions, ICDAS II scores, and age and gender of participants were recorded in a database under each patient's alphanumeric code.

### 2.3 Telediagnosis of dental caries

The photographs obtained were checked for sharpness, absence of dark areas, definition, and exposure by a second expert clinician (Clinician 2) using Microsoft Photos software (Microsoft Corporation). Zoom commands were also used where necessary to better focus the details of the images. Then only 5 of the 10 images (those with the best quality) were considered for the TD. Each tooth (except wisdom teeth) was assessed by Clinician 2 and the presence/absence of lesions, ICDAS II scores, and age and gender of participants were recorded in a database under each patient's alphanumeric code.

#### 2.3.1 Ethics

The study was conducted in accordance with the Declaration of Helsinki and the protocol was approved by the Review Board of the Section of Dentistry, Oral and Maxillofacial Surgery of the University of Verona. No ethics committee approval was required for this study, as already approved procedures were routinely performed.

All participants involved in the study were informed and signed a written informed consent form.

### 2.4 Statistical analysis

Descriptive statistics were conducted to analyze the demographic data (age and gender).

Photographs obtained from each participant were assessed for sharpness, absence of dark areas, and exposure and scored (0–5 points) for each parameter. Mann–Whitney tests were also performed to assess the differences between the photos taken with and without flash for each parameter. Statistical significance was set at ≤0.05.

In addition, statistical tests were performed as follows:

− Sensitivity of TD;
− specificity of TD;
− positive predictive value (PPV) of TD; and
− negative predictive value (NPV) of TD.

Spearman correlation to assess the relationship between the values obtained with TD and those obtained with CD was calculated. This correlation is an effect size and its strength can be described as follows: .00–.19 very weak; .20–.39 weak; .40–.59 moderate; .60–.79 strong; and .80–1.0 very strong.

All statistical tests were conducted using STATA16 software (StataCorp, 1985, California USA).

## 3 RESULTS

This clinical study involved 43 patients aged between 22 and 38 years (24.5 ± 2.7), 21 females and 22 males, and a total of 1201 teeth were analyzed.

A total of 430 photographs were sent in and examined, and no drop‐out was observed. No statistically significant differences were found between the photographs taken with and without flash for any parameter.

Only five photos per participant were used for telediagnosis of dental caries. The photos to be used were selected based on their better suitability to meet the aim of the study.

The good sensitivity of TD (74.0) was highlighted in the diagnosis of carious lesions and/or white spots. The specificity of TD was 99.1, indicating that TD is a highly specific method for identifying healthy tooth surfaces. The PPV of TD was 91.7 and the NPV of TD was 96.4. The Spearman's correlation was 0.816, indicating a very strong correlation between the values obtained with TD and those obtained with CD. The Spearman's correlation values showed a gradual underestimation of the ICDAS values from scores 2 to 4, indicating that telediagnosis is more effective in detecting initial enamel lesions than those advanced (Table 1).

**TABLE 1:** Correlation between ICDAS scores obtained with CD and TD

ICDAS score	TD‐ICDAS score 0	TD‐ICDAS score 2	TD‐ICDAS score 3	TD‐ICDAS score 4	Total
CD‐ICDAS score 0	1041	10	0	0	1051
	99.05%	0.95%	0.00%	0.00%	100%
CD‐ICDAS score 1	16	5	0	1	22
	72.72%	22.73%	0%	4.55%	100%
CD‐ICDAS score 2	16	59	1	1	77
	20.78%	76.62%	1.30%	1.30%	100%
CD‐ICDAS score 3	6	11	19	0	36
	16.67%	30.56%	52.79%	0.00%	100%
CD‐ICDAS score 4	1	3	5	6	15
	6.67%	20.00%	33.33%	40.00%	100%
Total	1080	88	25	8	1201
	89.93%	7.33%	2.08%	0.67%	100%

Abbreviations: CD, clinical diagnosis; ICDAS, International Caries Detection and Assessment System; TD, telediagnosis of dental.

## 4 DISCUSSION

The basis of this study was to evaluate the clinical feasibility of using remote diagnosis to detect carious lesions of the occlusal and vestibular surfaces of teeth.

It is conceivable that remote dental diagnosis is a feasible way for early diagnosis and improving patient adherence to appropriate health behaviors (Zotti et al., 2016; Zotti, Pietrobelli, et al., 2019; Zotti, Zotti, et al., 2019), as a large proportion of the population has a smartphone and these devices achieve high performance and quality images.

Telediagnosis of caries proved not to be as sensitive in the diagnosis of early‐stage enamel caries compared to CD, as the lesion has not changed its appearance significantly at this stage. On the other hand, it proved to be very accurate in diagnosing healthy teeth (specificity of 99.05%), while the sensitivity, i.e. the ability of the test to detect the disease, was lower (74.00%). The telediagnosis of initial carious lesions (ICDAS 2), with enamel cavitation (ICDAS 3) or more severe (ICDAS 4) agreed with the CD in 76.62%, 52.78%, and 40.00% of cases, respectively. This means that the correlation tends to decrease with decreasing severity of cavities.

This pattern and the decreasing sensitivity of telediagnosis could be due to several factors. First, it should be emphasized that the two diagnoses (TD and CD) were made by two different clinicians, and second, since the difference between ICDAS 2 and 3 is the presence of cavitation, the main motivation for the decrease in sensitivity could be found in the characteristics of the lesions.

It is impossible to assess the presence of cavitation from a photograph, so some lesions may be underestimated.

Telediagnosis of caries certainly cannot replace the clinical and radiographic diagnosis, which is always the gold standard for early and preventive detection of carious lesions. However, some considerations are needed when evaluating the benefits that can be achieved by using telediagnosis in caries diagnosis.

Considering that telediagnosis of dental caries might not be helpful in detecting hidden or minimal caries and interproximal caries and that these often require radiography, chairside visits, and radiography twice a year are essential for prevention and early diagnosis (Poorterman et al., 2000). Nevertheless, the opportunity to reduce the number of visits to the dentist represents a good opportunity from several points of view.

The benefits of telemedicine in terms of cost savings have been highlighted in the literature since 2004 (Johnston et al., 2004). However, this issue is controversial, especially due to the high initial costs of certain technologies required for certain diagnoses (Yoo et al., 2016). Several medical disciplines may indeed require software or specialized equipment for remote diagnosis, including cardiology or intensive care nursing (Yoo et al., 2018), and this circumstance could be a barrier to the spread of remote medicine. However, this issue is less significant in dentistry, where fewer technologies are required to take photographs of various oral conditions.

Recent literature highlights the importance of telemedicine and telediagnosis wherever possible. Recent studies (Sahin et al., 2021) have shown that telemedicine was highly valued by oncologists during the Covid 19 pandemic in Turkey, particularly the “stored and forward' method most commonly used in telediagnosis, which is also presented here.

Another critical issue in telematic caries diagnosis is the necessary requirement for taking appropriate photographs. In this study, the photographs did not always match the images in the protocol given to the patients. This is probably due to both the different levels of manual skills of the participants with the smartphones and the different quality of the smartphone cameras. Sometimes the surfaces of the teeth, especially those furthest from the lens (second molars), are not completely sharp or dark. In other cases, when observing the vestibular surfaces in the posterior areas of the premolar molars, the shadows in the interproximal areas could be mistaken for caries. For this reason, it seems important to look at photographs in both the flash and no flash versions to assess the differences.

Certainly, it is important to establish reproducible protocols and instruct patients on how best to use the tools in order to maximize the potential of telediagnosis and teledentistry.

Accordingly, the authors provided participants with a previously tested protocol for performing intraoral photographs so that all teeth, gingivae, and oral mucosa were visible and the technique of performing them was repeatable. To this end, the literature was not helpful as there were no preliminary or pilot studies that were ad hoc. Therefore, the protocol for taking photographs presented here is a pioneering proposal to provide a starting point on which improvements can follow.

Training patients to take photos correctly could be a useful way to minimize errors. Together with the protocol given to them, this would ensure that patients know the actual aim of the photos and are therefore more accurate when taking the photos or helping the person taking them.

We are aware that this is a weakness of this diagnostic method and that it could be improved, hopefully by improving the protocol for taking photos or by using different caries indices, or by improving the quality of the images and technologies. However, the predictable ability to diagnose healthy teeth could be an important advantage in terms of cost savings in a population with low decayed missed filled teeth (DMFt) and good oral hygiene compliance. This type of patient, based on these results, could reduce the number of dental visits while benefiting from the good sensitivity of remote diagnosis. This might represent an important plus for saving costs (travel, visits, disposables, professionals' time, patients' time, sterilization, risk in the dental office…) while ensuring preventive controls for patients.

Given the results, it may be interesting to increase the number of clinicians performing telediagnosis of caries to better understand the repeatability of this type of assessment and to highlight possible differences in the detection of the same lesions between users.

In addition, the teeth examined in this study had a low DMFt value, so it was not possible to assess multiple and extensive cavities. Therefore, it might be interesting to include a larger and more heterogeneous sample in the next studies to better test the sensitivity of telediagnosis of caries.

TD is not an exhaustive method for caries diagnosis and CD is essential. Nevertheless, TD could be a valid method to save time for regular check‐ups and costs.

With this in mind, further studies on the effectiveness of remote caries diagnosis would be desirable, especially those involving a larger sample. This would provide more reliable and reproducible results on both the diagnostic method and the clinician's skills in interpreting the images. In this context, it would be interesting to increase the number of clinical observers in the telematics phase to estimate the variability of interindividual interpretation.

## 5 CONCLUSION

The cross‐sectional observational clinical study performed applying the previously described protocol revealed the good potential of telediagnosis of caries (TD) which could be applicable on a large scale in the daily practice of preventive dentistry.

## AUTHOR CONTRIBUTIONS

Francesca Zottiand Francesco Simoncelli participated in the conception of the study, analysis, and interpretation of data, wrote the study, wrote the manuscript, critically reviewed the content, and approved the final version. Davide Pappalardoparticipated in the conception of the study, critically reviewed the content, and approved the final version. Luca Rosolin participated in the conception of the study, in the analysis and interpretation of data, wrote the manuscript, critically reviewed the content, and approved the final version. Annalisa Cominziolli and Nicoletta Zerman participated in the conception of the study and approved the final version.

## CONFLICT OF INTEREST

The authors declare no conflict of interest.

## Data Availability

Research data are not shared.
